# Mental Deficiency (Amentia)

**Published:** 1909-06

**Authors:** 


**Mental Deficiency (amentia).** By A. F. Tredgold. Pp. xviii, 391. London : Baillière, Tindall & Cox. 1908.

Dr. Tredgold has contributed an excellent addition to the literature dealing with Mental Deficiency. His book in great part will repay perusal by the medical profession in general. He is well qualified to write upon this subject, and has accumulated abundant and valuable material based upon personal research. Throughout the book reads concisely and clearly. Where necessary the investigations of other workers are quoted and suitably acknowledged, but in the main the production is marked by originality of thought and independent labour. We proceed to furnish some quotations from the book. In dealing with the causation of amentia, Dr. Tredgold adopts a classification of "Intrinsic and Extrinsic." By the former is implied influences modifying the germinal plasm prior to conception ; by the latter (or environmental influences) those affecting development whilst within or without the uterus. It will be seen that his intrinsic group has a more limited definition than has the ordinary congenital class of cases. Instances of true primary amentia form 90 per cent. of the total. The author does not endorse the theory of Weismann. He finds it impossible to avoid the conclusion that a serious deterioration of germ-plasm may be brought about by disturbance of metabolism, notably to be occasioned by alcoholism and phthisis in the parents. His views expressed on the course of degeneration are clear, decisive and based on a personal investigation and compilation both careful and ingenious. He regards the accumulation and intermarriage of derelict's residual in country districts as the chief cause of the rural disproportion of aments; whilst in the great centres of population the effects of unhealthy environment, drink, phthisis, &c., tend rather to insanity in the first instance, although amentia results later as a development of this.

The class of secondary (or extrinsic) aments is a small one, some 10 per cent. of the whole. In these defect is brought about by general or localised disease of nerve cells or by external factors influencing their nutrition. In this group are included lesions vascular, inflammatory and toxic in nature. The essential basis of amentia is an imperfect or arrested development of the central neurons, as well as a numerical deficiency. Embryonic cells are found in undue number in the second and third pyramidal layers of all regions of the cortex, but mainly in the prefrontal and parietal lobes. But added and grosser pathological lesions may complicate primary amentia, or form the sole cause of secondary defect. In proportion to the extent of these and their progressive tendency, be it here remarked, is the prospect of improvability. A considerable proportion of cases of secondary amentia is less capable of this than those of primary kind, owing to the advancing nature of the cerebral lesions. The author remarks that too much stress should not be laid upon physical stigmata as indicative of mental degeneration; it is needful to differentiate those due to inherent defects of germ-plasm from those produced by secondary influences. The inherent "blight" of the primary form gives rise to numerous and widespread anomalies of anatomical development absent in the secondary form. The secondary ament is often well-grown and developed, or with occasional abnormality due to some particular lesion present. Dr. Tredgold combats the view that where paralysis results from this mental deficiency is a necessary co-relate. He has seen many cases in which extensive limb paralysis existed with unimpaired intelligence. The prognosis is less favourable where epilepsy is a complication. An interesting hypothesis is advanced to account for instances of supposed compensation after a cerebral deprivation; that is, there is normally an undeveloped or potential reserve of embryonic neuroblasts which under certain conditions may take on active evolution, and replace nerve cells destroyed by a lesion on the same, perhaps even on the opposite side of the brain. In regard to syphilis, Dr. Tredgold finds that cases of amentia attributable to it alone form but a half per cent. of the total, and including neurotic heritage, the number reaches but one to two per cent. Its action he considers to be by general dystrophic arrest of neuronic growth, a rare event; usually by direct poisoning of developing cortical cells. Anti-treatment has proved of no avail.

There are excellent chapters dealing with the clinical varieties of amentia, social and legal relations of the defectives, and the potentialities and methods of treatment and training. The work is well printed and illustrated by typical photographs of cases.

